# Clopidogrel Protects Endothelium by Hindering TNF*α*-Induced VCAM-1 Expression through CaMKK*β*/AMPK/Nrf2 Pathway

**DOI:** 10.1155/2016/9128050

**Published:** 2015-12-28

**Authors:** Huabing Yang, Pengjun Zhao, Shiliu Tian

**Affiliations:** ^1^School of Medical Sciences, Hubei University of Chinese Medicine, Wuhan 430065, China; ^2^Department of Medicine and Harold Hamm Oklahoma Diabetes Center, University of Oklahoma Health Sciences Center, Oklahoma City, OK 73104, USA; ^3^Pediatric Heart Center, Children's Hospital of Fudan University, Shanghai 200032, China; ^4^Key Laboratory of Exercise and Health Sciences of Ministry of Education, Shanghai University of Sport, Shanghai 200438, China

## Abstract

Clopidogrel (INN), an oral antiplatelet drug, has been revealed to have a number of biological properties, for instance, anti-inflammation and antioxidation. Oxidative stress plays an imperative role in inflammation, diabetes mellitus, atherosclerosis, and cancer. In the present study, human aortic endothelial cells (HAECs) were employed to explore the anti-inflammatory activity of INN. INN reduced TNF*α*-induced reactive oxygen species (ROS) generation and time-dependently prompted the expression and activity of heme oxygenase 1 (HO-1). Cellular glutathione (GSH) levels were augmented by INN. shHO-1 blocked the INN suppression of TNF*α*-induced HL-60 cell adhesion. The CaMKK*β*/AMPK pathway and Nrf2 transcriptional factor were implicated in the induction of HO-1 by INN. Additionally, TNF*α* dramatically augmented VCAM-1 expression at protein and mRNA levels. INN treatment strikingly repressed TNF*α*-induced expression of VCAM-1 and HL-60 cell adhesion. Compound C, an AMPK inhibitor, and shNrf2 abolished TNF*α*-induced expression of VCAM-1 and HL-60 cell adhesion. Our data suggest that INN diminishes TNF*α*-stimulated VCAM-1 expression at least in part via HO-1 induction, which is CaMKK*β*/AMPK pathway-dependent.

## 1. Introduction

Inflammation is common for patients with cardiovascular diseases and is considered to be a sign or atherogenic response. Reactive oxygen species (ROS) such as superoxide anions, hydrogen peroxide, peroxynitrite, and hydroxyl radicals plays an important role in inflammation, leading to endothelial oxidative damage and dysfunction of the cardiovascular system. Proinflammatory cytokines, for example, tumor necrosis factor-alpha (TNF*α*), are able to stimulate ROS liberation [1]. Superfluous ROS production not only causes endothelial dysfunction [2] but also stimulates signaling transduction pathways implicated in augmented gene expression of inflammation-related cytokine [3]. In cardiovascular cells, the normal intracellular ROS levels rely on the proper balance between ROS formation and antioxidant defense systems.

Sophisticated interactions between leukocytes and the endothelium are involved in inflammatory reaction. The endothelial cell (EC) surface is relatively smooth and nonadhesive. In cardiovascular diseases, the interactions between the ECs and components of the blood are altered by adhesion molecules, for example, ICAM-1 and VCAM-1 [4]. The endothelial cells are activated, leading to overexpression of adhesion molecules at inflammation sites. The proinflammatory molecule TNF-*α* prominently elevates adhesion molecules on the endothelium [5–7]. Additionally, substantial evidence demonstrates an augmented expression of VCAM-1 in inflammatory animal models and human atherosclerotic plaques [8].

Heme oxygenase 1 (HO-1) possesses antioxidant properties [9]. Its key function is to degrade heme to iron, carbon monoxide (CO), and biliverdin [10, 11]. Biliverdin, via the cytosolic enzyme biliverdin reductase, is converted to bilirubin, which possesses antioxidant characteristics [12]. In addition, HO-1-derived CO is involved in vasoregulation and signal transduction [13, 14]. Some signaling molecules, for instance, 5′ adenosine monophosphate-activated protein kinase (AMPK), mitogen-activated protein kinases (MAPK), and PI3K/Akt, as well as transcriptional factors including activator protein 1 (AP-1) and NF-E2-related factor-2 (Nrf2), regulate HO-1 gene expression [15–18].

Clopidogrel (INN) is an oral antiplatelet drug hindering blood clots and has been discovered to possess several biological properties such as anti-inflammation, antioxidation, and antiatherosclerosis [19–21]. Anti-inflammation and antioxidation can be used as therapeutic strategies to prevent or treat cardiovascular diseases. The present study was carried out to elucidate the role of AMPK and HO-1 in the INN inhibition of TNF*α*-stimulated expression of VCAM-1 and the underlying mechanisms implicated.

## 2. Materials and Methods 

### 2.1. Reagents

Primary human aortic endothelial cells (HAECs) and endothelial growth factors were purchased from Cambrex Bioscience (Rockland, ME). HAECs were cultured in M199 complete medium. Medium 199, gentamycin, fungizone, glutamine, collagenase, gelatin, trypsin/EDTA, penicillin, and streptomycin were purchased from Sigma-Aldrich (St. Louis, MO). Fetal bovine serum (FBS) was purchased from HyClone (Logan, UT, USA). HL-60 cells were cultured in Roswell Park Memorial Institute (RPMI) 1640 plus l-glutamine and 25 mM HEPES supplemented with 15% FBS [22]. Human TNF*α*, DMSO, HEPES, sodium bicarbonate, Compound C, and all other chemicals were from Sigma. STO-609 was purchased from Tocris (Ellisville, Missouri). Clopidogrel (INN) (Plavix, Sanofi-Aventis, BN.F-33565, France) stock solution (10 mM) was prepared by dissolving it in DMSO. TRIzol RNA extraction reagent and 2,7-dichlorofluorescin diacetate (H2DCF-DA) were purchased from Invitrogen (Carlsbad, CA, USA). Antibodies reacting with AMPK and phosphor-AMPK (Thr172) were obtained from Cell Signaling Technology (Danvers, MA); antibodies reacting with *β*-actin and Nrf2 were obtained from Santa Cruz Biotechnology (Santa Cruz, CA); anti-HO-1 and VCAM-1 antibodies were obtained from Abcam (Cambridge, MA, USA). Cell culture HAECs were cultured in medium 199 added with 20 mM HEPES, 2 mM glutamine, 20% FBS, 100 mU/L penicillin, and 100 mg/L streptomycin at 37°C. The medium was refreshed every 48 h until confluence. Preincubation with inhibitors was implemented in HEPES-HSA buffer (10 mM HEPES, 10 mM glucose, 1.5 mM CaCl_2_, 145 mM NaCl, 5 mM KCl, 1 mM MgSO_4_, and 0.25% HSA). Compound C and STO-609 were dissolved in dimethyl sulfoxide (DMSO).

### 2.2. Western Blotting

ECs were lysed in the lysis buffer. Equivalent amounts of protein samples were separated by SDS-PAGE and then transferred to nitrocellulose membrane followed by immunoblotting with the primary antibodies including phosphor-AMPK-Thr172, AMPK, HO-1, Nrf2, PARP, and *β*-actin.

### 2.3. Real-Time PCR

Total RNA was extracted by using a QIAshredder column and RNeasy kit (Qiagen) and frozen at −80°C for further RT-PCR analysis. Primer sequences were as follows: *β*-actin (ATGTTTGAGACCTTCAACAC, CACGTCACACTTCATGATGG), VCAM-1 (CGTCTTGGTCAGCCCTTCCT, ACATTCATATACTCCCGCATCCTTC), and HO-1 (GGGTGATAGAAGAGGCCAAGA, AGCTCCTGCAACTCCTCAAA). cDNA (1 *μ*L) was amplified in PCR solution (25 *μ*L) in a Mini OpticonTM Real-Time PCR Detection System. Cycle parameters were set as 95°C for 15 min, 40 cycles of 95°C for 15 sec, 58°C for 1 min, and 72°C for 1 min.

### 2.4. HO-1 Activity Assay

HO-1 activity was determined as described previously [23]. The HO-1 activity in EC lysates was calculated as picomoles bilirubin produced per hour per milligram of total protein (pmol BR h-1 mg-1) and the data were expressed as fold HO-1 activity compared to control cells.

### 2.5. Subcellular Fractionation

Subcellular fractionation was performed with a Subcellular Protein Fractionation Kit (Thermo Fisher Scientific Inc., Rockford, IL, USA) according to the manufacturer's protocol. Nrf2 protein levels in the nuclear fractions were measured by Western analysis. Expression of PARP was utilized as loading controls for the purity of the nuclear extracts.

### 2.6. RNA Interference with shRNA

Lentiviral infection was implemented as described previously [24]. Two distinct sequences targeting human Nrf2 and HO-1 mRNA were selected and obtained from Sigma (St. Louis, MO, USA). The shRNA sequences were as follows: Nrf2 shRNA #1, 5′-GCTCCTACTGTGATGTGAAAT-3′, shRNA #2, 5′-CCGGCATTTCACTAAACACAA-3′; HO-1 shRNA #1, 5′-GCTGAGTTCATGAGGAACTTT-3′, shRNA #2, 5′-GCTGAGTTCATGAGGAACTTT-3′; shLuc shRNA, 5′-CAAATCACAGAATCGTCGTAT-3′. shLuc was used as a vector control.

### 2.7. Reactive Oxygen Species (ROS) Measurement

Intracellular ROS state was determined with the cell-permeant 2′,7′-dichlorodihydrofluorescein diacetate (H2DCFDA) as previously described [25]. Concisely, HAECs were grown to 50% confluence followed by serum starvation in medium 199 supplemented with 0.5% (v/v) FBS for another 24 h. The ECs were maintained in serum-free medium without phenol red for 15 min preceding being exposed to TNF*α*. ECs were incubated with H2DCFDA (10 *μ*M) for 10 min and instantly observed under a confocal microscope (Leica TCS SP2).

### 2.8. Cellular GSH Assay

Cellular GSH assay was performed as described previously [26]. Briefly, ECs were washed two times with ice cold PBS. The homogenate was prepared in the potassium phosphate buffer (20 mM, pH 7.0) and centrifuged at 10,000 ×g for 20 min at 4°C. The supernatant was kept as the cell lysate. The protein was measured with a BCA Protein Assay Kit. Cell lysates (100 *μ*L) were incubated with 5% TCA (150 *μ*L) and centrifuged at 5000 ×g for 10 min at 4°C. The cell lysate was incubated with 0.4 M Tris buffer and 0.01 M DTNB. After incubation at room temperature for 5 min, the intracellular GSH production was measured with a microplate reader at 412 nm (Model 680, Bio-Rad).

### 2.9. Monocyte Adhesion Assay

The monocyte adhesion assay was executed as described by Chen et al. [27].

### 2.10. Statistical Analysis

The results were expressed as mean ± SD. Data were analyzed with one-way analysis of variance and Fisher's protected least significant difference test. *P* < 0.05 was considered as the level of significance.

## 3. Results 

### 3.1. Effect of Clopidogrel (INN) on TNF*α*-Induced ROS Formation in HAECs

**Figure 1:**
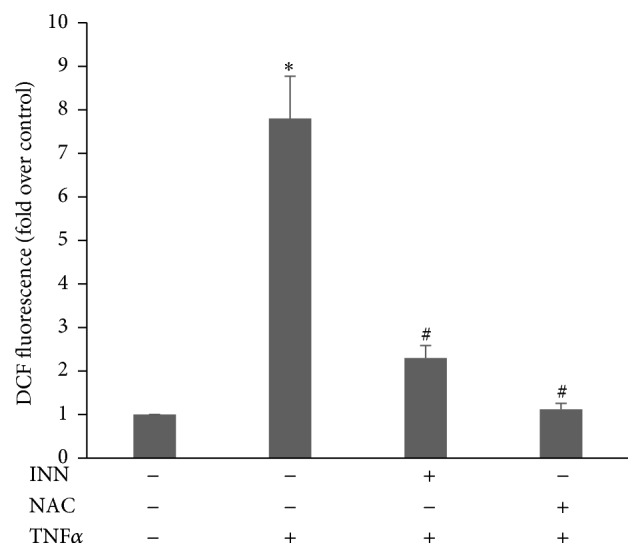
Effect of Clopidogrel (INN) on TNF*α*-stimulated ROS liberation in HAEC. ECs were pretreated with 10 *μ*M INN for 24 h or 1 mM NAC for 2 h and treated with 10 mM H2DCFDA for 15 min prior to being challenged with TNF*α* (1 ng/mL) for another 20 min. Cells incubated with 0.1% DMSO for 24 h were used as a control (CON). ROS levels were assayed with a BioTek Fluorescence Plate Reader. The mean DCF value of control is set as “1.” The relative fluorescence intensity is presented as mean ± SD. ^*∗*^ *P* < 0.05 (*n* = 3, Student's *t*-test).

To determine whether INN decreases TNF*α*-stimulated ROS formation, we pretreated ECs with 10 *μ*mol/L INN for 24 h and then treated ECs with 1 ng/mL TNF*α* for another 20 min. As illustrated in Figure 1, TNF*α* promoted ROS liberation at 20 min, and pretreatment with 10 *μ*mol/L INN dramatically repressed this ROS liberation. N-Acetylcysteine (NAC) is a potent antioxidant and used as a positive control.

### 3.2. INN Enhances HO-1 Expression and Hinders TNF*α*-Stimulated HL-60 Adhesion

**Figure 2:**
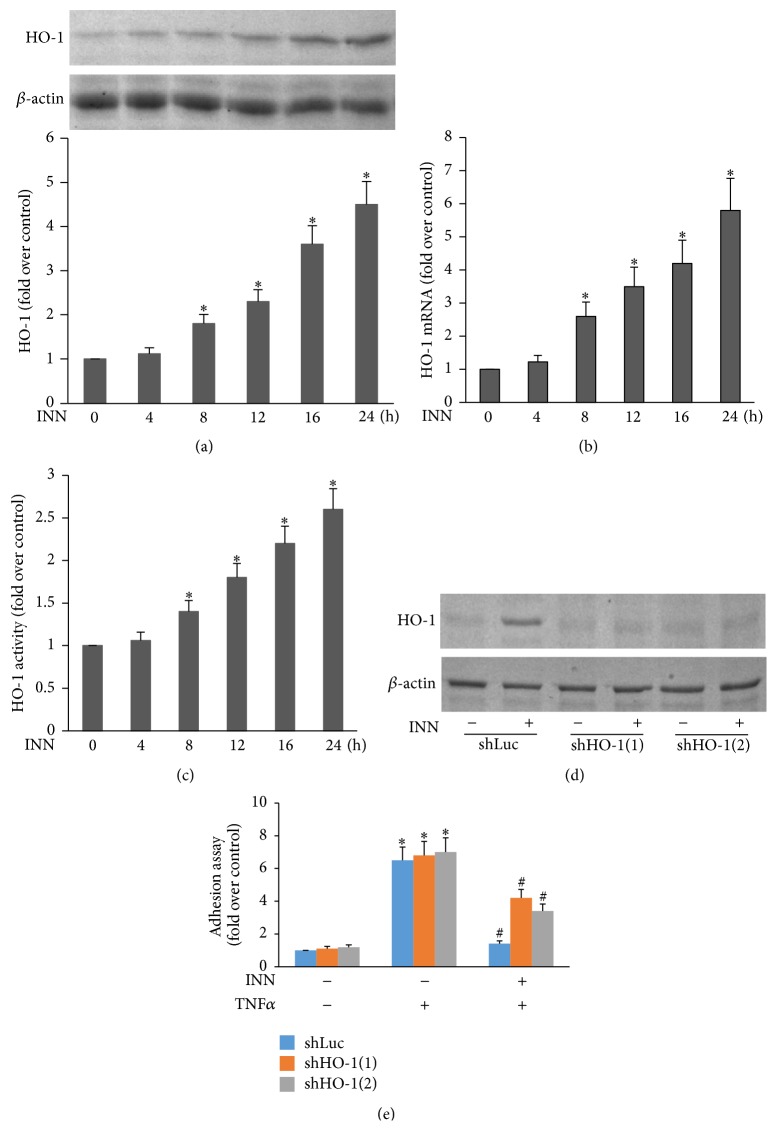
The role of HO-1 in INN suppression of TNF*α*-triggered HL-60 cell adhesion. ECs were incubated with 10 *μ*M INN for the indicated time points. Aliquots of lysate (50 *μ*g) were subjected to Western analysis for HO-1 protein expression (a). Total RNA was extracted from ECs and underwent real-time PCR with specific primers for HO-1 and *β*-actin (b). HO-1 activity was measured three times in each treatment group as mentioned in Methods. Data are expressed as fold increase of HO-1 activity versus control (c). ECs transfected with shHO-1 were incubated with 10 *μ*M INN for 24 h prior to being stimulated with 1 ng/mL TNF*α* for additional 6 h (d). (e) ECs were incubated with 1 ng/mL TNF*α* in the presence or absence of INN for 8 h. Adhesion assay was performed as described in Materials and Methods. Values are means ± SD (*n* = 4). ^*∗*^ *P* < 0.05 versus control; ^#^ *P* < 0.05 versus TNF*α*.

Since HO-1 has a potent cytoprotective effect against cellular oxidative stress [28, 29], we sought to investigate the effect of INN on HO-1 expression. HAECs were incubated with 10 *μ*M INN for various time periods. As illustrated in Figures 2(a), 2(b), and 2(c), INN treatment time-dependently promoted expression of HO-1 at mRNA and protein levels as well as its activity. Furthermore, the role of HO-1 in the suppression of HL-60 adhesion by INN was determined with HO-1 shRNA knockdown study. ECs were transfected with shHO-1, followed by incubation with 10 *μ*M INN for 24 h and then ECs were stimulated with TNF*α* for another 6 h. Knockdown of HO-1 was confirmed by Western blot (Figure 2(d)). As indicated in Figure 2(e), shHO-1 lessened the INN suppression of HL-60 adhesion. Together, our data suggest that HO-1 plays an imperative role in the suppression of TNF*α*-stimulated HL-60 adhesion by INN.

### 3.3. The CaMKK*β*/AMPK Pathway Mediates INN-Induced HO-1 Expression

**Figure 3:**
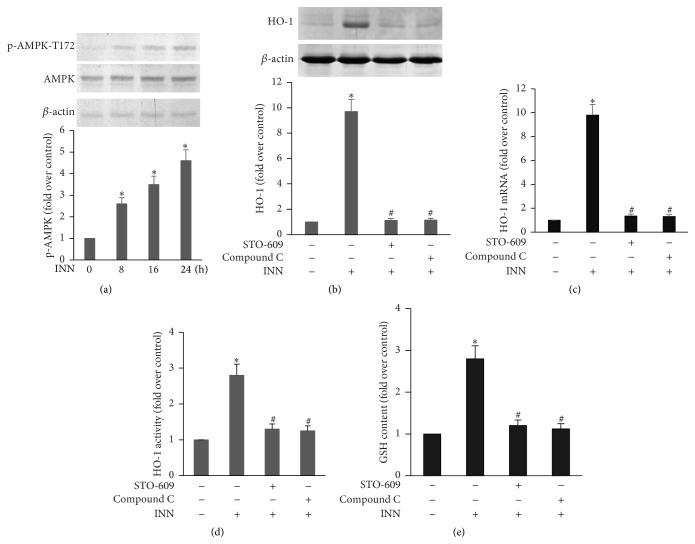
CaMKK*β*/AMPK is involved in INN-induced HO-1 expression and GSH synthesis. ECs were incubated with 10 *μ*M INN for various time periods. (a) Total protein was subjected to Western analysis for p-AMPK and AMPK expression. ECs were treated with 10 *μ*M STO-609 and Compound C for 1 h, followed by incubation with 10 *μ*M INN for another 16 h. Aliquots of cell lysate underwent immunoblotting analysis for HO-1 expression (b). Total RNA was extracted and underwent RT-PCR with specific primers for HO-1 and *β*-actin (c). HO-1 activity was measured three times in each treatment group as mentioned in Methods. Data are expressed as fold increase of HO-1 activity versus control (d). ECs were treated with 10 *μ*M STO-609 and Compound C for 1 h, followed by incubation with 10 *μ*M INN for another 24 h. GSH content was detected as mentioned in Methods (e). The results represent means ± SD (*n* = 3). ^*∗*^ *P* < 0.05 versus control; ^#^ *P* < 0.05 versus INN.

Previous studies [30] suggest that AMPK modulates the antioxidant status of cardiovascular ECs by upregulating expression of genes implicated in antioxidant defense, for example, manganese superoxide dismutase, catalase, and thioredoxin. To determine the signaling pathways implicated in the INN-mediated HO-1 induction, we examined the phosphorylation of AMPK. ECs were incubated with INN (10 *μ*M) for different time periods. As illustrated in Figure 3(a), AMPK was activated by INN. However, INN did not change AMPK protein expression. To further substantiate the role of the CaMKK*β*/AMPK pathway, their specific inhibitors, STO-609 and Compound C, were used. As shown in Figures 3(b), 3(c), and 3(d), STO-609 and Compound C attenuated INN-triggered HO-1 expression and activity. As indicated in Figure 3(e), the INN-stimulated elevation of cellular GSH content was repressed by STO-609 and Compound C. Our findings demonstrate that INN induces HO-1 expression and increases GSH synthesis through the CaMKK*β*/AMPK pathway.

### 3.4. Nrf2 Is Activated by INN and Implicated in the HO-1 Induction

**Figure 4:**
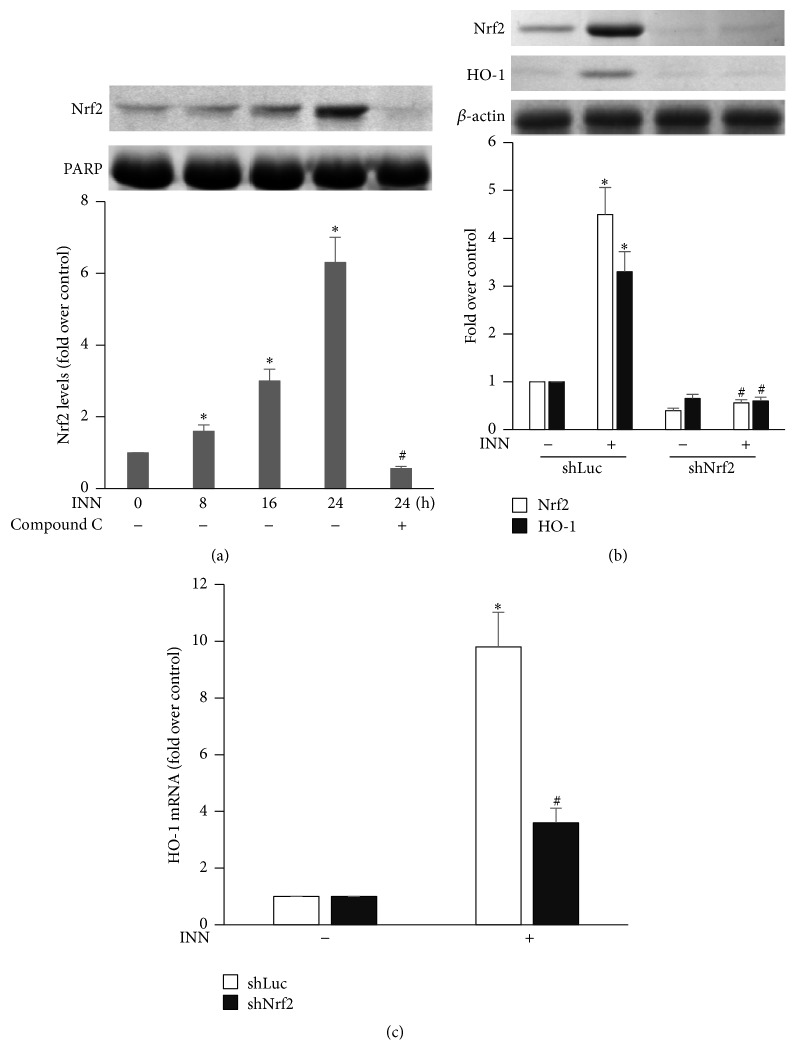
Nrf2 are implicated in HO-1 expression induced by INN. (a) ECs were treated with 10 *μ*M Compound C for 1 h followed by incubation with 10 *μ*M INN for the indicated time. Aliquots of cell lysate underwent immunoblotting analysis. ^*∗*^ *P* < 0.05 versus control; ^#^ *P* < 0.05 versus INN (24 h). (b) ECs were transfected with shLuc or shNrf2 and then treated with 10 *μ*M INN for 16 h. Aliquots of cell lysate underwent immunoblotting analysis. (c) Total RNA was extracted from ECs and was used to analyze HO-1 and *β*-actin mRNA by using RT-PCR with their specific primers. Data are expressed as means ± SD (*n* = 3). ^*∗*^ *P* < 0.05 versus control; ^#^ *P* < 0.05 versus INN/shLuc.

To illuminate the downstream signaling pathway of CaMKK*β*/AMPK for the stimulation of HO-1 by INN, we examined Nrf2 nuclear translocation. ECs were incubated with 10 *μ*M INN for the indicated time. As indicated in Figure 4(a), Nrf2 nuclear translocation was augmented as early as 8 h and continued until 24 h after INN treatment. Furthermore, Nrf2 nuclear translocation was repressed in ECs pretreated with the AMPK inhibitor Compound C for 1 h before being challenged with 10 *μ*M INN. Our results suggest that the CaMKK*β*/AMPK pathway is implicated in INN-stimulated Nrf2 nuclear translocation. To further determine the role of Nrf2 in INN-stimulated HO-1 expression, ECs were transfected with shNrf2 and shLuc. As illustrated in Figures 4(b) and 4(c), knockdown of Nrf2 eliminated INN-induced HO-1 expression. Together, our results demonstrate that Nrf2 is a transcriptional factor responsible for INN-stimulated HO-1 expression.

### 3.5. AMPK and Nrf2 Are Responsible for INN Inhibition of TNF*α*-Stimulated VCAM-1 Expression and Monocyte Adhesion in HAEC

**Figure 5:**
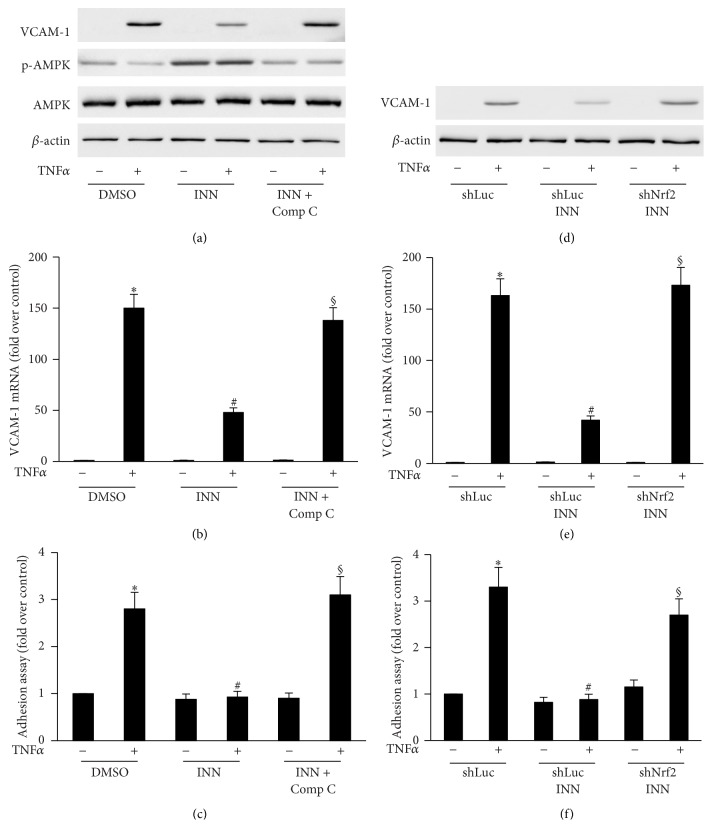
Effect of INN alone or in combination with AMPK or Nrf2 inhibition on TNF*α*-stimulated VCAM-1 expression and monocyte adhesion in HAEC. (a) ECs were treated with 1 ng/mL TNF*α* with or without DMSO (vehicle), INN, or INN + Compound C (Comp C) for VCAM-1, p-AMPK, and AMPK expression measurement. (d) ECs transfected with shLuc or shNrf2 were incubated with 1 ng/mL TNF*α* in the presence or absence of INN for 8 h for VCAM-1 expression measurement. Aliquots of cell lysate underwent immunoblotting analysis. (b and e) Total RNA was extracted and underwent RT-PCR with specific primers for VCAM-1. (c and f) Adhesion assay was carried out as described in Methods. Results are expressed as means ± SD (*n* = 3). ^*∗*^ *P* < 0.05 versus control; ^#^ *P* < 0.05 versus TNF*α*; ^§^ *P* < 0.05 versus TNF*α*/INN.

Next, we sought to determine whether INN hinders TNF*α*-induced VCAM-1 activation. As indicated in Figure 5, TNF*α* significantly increased expression of VCAM-1 and HL-60 cell adhesion. INN treatment dramatically suppressed TNF*α*-induced VCAM-1 expression at mRNA and protein levels as well as HL-60 cell adhesion. To further identify the role of AMPK and Nrf2 in the TNF*α*-stimulated VCAM-1 expression, we then used Compound C to inhibit AMPK and shNrf2 to knock down Nrf2 expression. As illustrated in Figure 5, Compound C and shNrf2 abolished TNF*α*-triggered VCAM-1 expression and HL-60 cell adhesion. Together, these results suggest that AMPK and Nrf2 mediate INN inhibition of TNF*α*-stimulated VCAM-1 expression and monocyte adhesion in HAEC.

## 4. Discussion 

Improvement of intracellular antioxidant ability is thought to diminish the risk of oxidative stress-induced diseases. Clopidogrel (INN) is an oral antiplatelet drug used to impede blood clots in coronary artery disease (CAD). Previous studies have shown that INN has anti-inflammation and antioxidation activities [19–21]. Recent work has demonstrated that INN augments nitric oxide (NO) and prostacyclin production in endothelial cells [31]. In addition, INN impedes CD40 ligand both in vitro and in vivo [32], stimulating HO-1 expression [33]. These findings suggest that INN can preserve endothelial function via a mechanism independent of its antiplatelet activity. More importantly, McClung et al. suggest that INN protects against diabetes-induced vascular damage and reduces circulating endothelial cells (CECs) in type-2 diabetes patients [34]. The effect of INN was to improve vascular function, protect against oxidative stress, and inhibit apoptosis in patients with type-2 diabetes. This involved an increase in the expression of both phosphorylated Akt and AMPK. In the present study, we revealed that INN repressed TNF*α*-stimulated ROS liberation, VCAM-1 expression, and cell adhesion and that this effect was related to the increase in HO-1 expression and GSH content via the CaMKK*β*/AMPK/Nrf2 pathway.

TNF*α* is a powerful proinflammatory cytokine secreted by various innate immune cells, predominantly activated macrophages, as well as neutrophils, mast cells, and eosinophils cells [35–39]. In macrophages, inflammatory stimuli result in TNF*α* synthesis and release by constitutive exocytosis [38]. The plasma levels of TNF*α* are augmented in some pathologies, such as cancer, atherosclerosis, rheumatoid arthritis, and preeclampsia [40]. Several studies have revealed that TNF*α*, via activation of NF*κ*B, induces expression of proinflammatory cytokines and adhesion molecules, for example, IL-6 and VCAM-1 [41, 42]. In the current study, we revealed that TNF*α* increased ROS production, VCAM-1 expression, and cell adhesion. Intriguingly, these effects of TNF*α* were abolished by INN.

Heme oxygenase or haem oxygenase (HO) is a stress-inducible enzyme. Increasing evidence supports that it protects against many chronic diseases such as cardiovascular diseases, hypertension, diabetes mellitus, and neurological disorders [43]. Using systemic manipulation of either HO-1 expression or activity, some studies suggested that HO-1 plays a vital role in atherosclerosis initiation and development. HO-1-null mice show a noteworthy increase in plasma lipid hydroperoxides [44]. Similarly, rabbits treated with SnPP, an HO-1 inhibitor, exhibit a significant lipid deposits in abdominal aortic plaques [45]. Contrarily, HO-1 induction alleviated the oxLDL-induced formation of foam cells [46]. Taken together, these studies substantiated the anti-inflammatory or antiatherosclerotic activity of HO-1.

The early step in atherogenesis is endothelial dysfunction resulting in several compensatory responses that change the vascular homeostasis [47]. Proinflammatory stimuli such as a diet rich in saturated fat, obesity, hypercholesterolemia, and hyperglycemia cause the expression of adhesion molecules, for example, vascular cell adhesion molecule-1 (VCAM-1) and P-selectin in endothelium, and these molecules facilitate the attachment of monocytes and lymphocyte [48, 49]. In addition, turbulent flow resulting from an unfavorable serum lipid profile probably leads to overexpression of adhesion molecules in endothelial cells triggering atherosclerosis. Animals fed a proatherogenic diet quickly overexpress VCAM-1 [50]. Overexpression of VCAM-1 enhances recruitment of monocytes to endothelial injury locations; succeeding liberation of monocyte chemoattractant protein-1 (MCP-1) by leukocytes amplifies the inflammatory cascade through recruiting other leukocytes, stimulating leukocytes in the media, and initiating recruitment and proliferation of smooth muscle cells [51]. Here, we demonstrate that suppression of VCAM-1 expression by INN reduced HL-60 cell adhesion to TNF*α*-stimulated HAECs. Nevertheless, this inhibition was eliminated by AMPK inhibitor Compound C, shHO-1, and shNrf2 (Figures 2(d), 5(c), and 5(f)). These data demonstrate the contribution of AMPK, Nrf2, and HO-1 to the suppression of HL-60 cell adhesion by INN.

Nrf2 is an imperative transcriptional factor implicated in cellular anti-inflammatory action and related to the induction of HO-1 and glutathione S-transferase [52, 53]. Nrf2 interacts with its cytosolic inhibitor Keap1 under basal circumstances. In response to stress, Nrf2 is released from Keap1 and successively translocates to the nucleus, activating its target gene transcription via ARE [54]. In the current study, INN augmented Nrf2 nuclear translocation, and silencing Nrf2 abolished the INN induction of HO-1. These results indicate that this transcriptional factor is indispensable for INN-induced HO-1 expression. Nrf2 activation is controlled by several kinases, including JNK, p38, ERK, and PI3K/Akt [55]. In the current study, we demonstrated that INN activates CaMKK*β* and AMPK. By using specific inhibitors of CaMKK*β* and AMPK, we revealed that the CaMKK*β*/AMPK pathway is implicated in the INN-triggered HO-1 induction.

**Figure 6:**
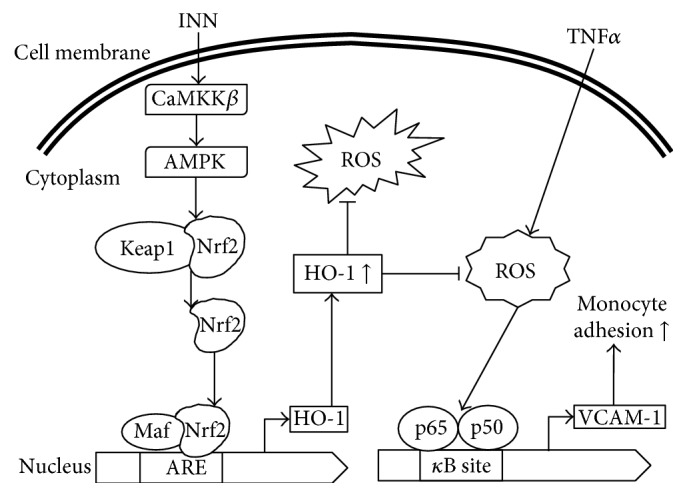
Proposed diagram summarizing the suppression of TNF*α*-induced inflammation by INN via upregulating HO-1 expression through the CaMKK*β*/AMPK/Nrf2 pathway and repressing ROS formation, VCAM-1 expression, and monocyte adhesion. In summary, we have revealed that INN hinders TNF*α*-induced ROS formation, expression of VCAM-1, and HL-60 cell adhesion by upregulating HO-1 gene expression and elevating GSH levels through the CaMKK*β*/AMPK/Nrf2 pathway.

In summary, we have revealed that INN hinders TNF*α*-induced ROS formation, expression of VCAM-1, and HL-60 cell adhesion by upregulating HO-1 gene expression and elevating GSH levels through the CaMKK*β*/AMPK/Nrf2 pathway (Figure 6). The antioxidant and anti-inflammatory characteristic of INN is thought to protect against oxidative stress-induced diseases including arthrosclerosis.
